# Environmental Footprint of Antibiotics: A Multi-Source Investigation of Wastewater Systems in UAE

**DOI:** 10.3390/antibiotics14111105

**Published:** 2025-11-02

**Authors:** Shahana Seher Malik, Balamurugan Sadaiappan, Ashraf Aly Hassan, Iltaf Shah, Sampathkumar Elangovan, Sunil Mundra

**Affiliations:** 1 Department of Biology, College of Science, United Arab Emirates University, Al Ain P.O. Box 15551, United Arab Emirates; 202090120@uaeu.ac.ae (S.S.M.); balamurugan.s@uaeu.ac.ae (B.S.); 2 Khalifa Center for Genetic Engineering and Biotechnology, United Arab Emirates University, Al Ain P.O. Box 15551, United Arab Emirates; 3 Department of Civil and Environmental Engineering, College of Engineering, United Arab Emirates University, Al Ain P.O. Box 15551, United Arab Emirates; alyhassan@uaeu.ac.ae; 4 Department of Chemistry, College of Science, United Arab Emirates University, Al Ain P.O. Box 15551, United Arab Emirates; altafshah@uaeu.ac.ae (I.S.);; 5 National Water and Energy Center, United Arab Emirates University, Al Ain P.O. Box 15551, United Arab Emirates

**Keywords:** antibiotics, hospital, LC-MS/MS, residential community, SPE, wastewater

## Abstract

**Background/Objectives**: Antibiotic resistance is a growing global health threat, requiring comprehensive investigations into the occurrence, distribution, and diversity of antibiotics across environmental systems. This study aimed to examine the distribution and prevalence of commonly used antibiotics in various wastewater sources across the United Arab Emirates (UAE), providing insights into potential environmental reservoirs contributing to antimicrobial resistance dissemination. **Methods**: Wastewater samples were collected from the outlets of three hospitals, three residential communities, and the influent and return activated sludge of two wastewater treatment plants. Samples were prepared using solid-phase extraction (SPE) and analyzed via liquid chromatography–mass spectrometry (LC–MS/MS) to quantify antibiotic concentrations and compare their distribution among sources. **Results**: Significant variations were observed in antibiotic concentrations across the different wastewater sources. Ciprofloxacin concentrations were highest in hospital wastewater, reaching up to 247.9 ng/mL, while cefuroxime and vancomycin ranged between 0.2 and 10.9 ng/mL across samples. Clindamycin was notably prevalent in wastewater treatment plant samples (up to 10.9 ng/mL), whereas vancomycin and cefuroxime were dominant in residential community samples, reflecting diverse antibiotic usage patterns and persistence in treatment systems. **Conclusions**: The findings reveal substantial antibiotic contamination in United Arab Emirates wastewater systems, underscoring the need for ongoing surveillance and regulatory measures. Continuous monitoring, coupled with effective wastewater treatment and antibiotic stewardship strategies, is critical to mitigating the environmental spread of antimicrobial resistance and safeguarding public and environmental health.

## 1. Introduction

In recent years, pharmaceuticals have garnered significant interest due to their role in environmental challenges, with antibiotics holding particular importance. Since their discovery in 1920, antibiotics have been extensively used to combat human and veterinary diseases by either killing or inhibiting bacterial growth [1,2]. However, concerns have arisen regarding their excessive or unnecessary usage, leading to the prevalence of antibiotic resistance (ABR), escalating future treatment costs, and potentially rendering certain infections incurable [3]. Frequently, antibiotics administered to humans and animals are not fully metabolized, with 90% remaining active after administration. Consequently, majority of these substances or their metabolites are expelled from the body through urine and feces, either unchanged or partially altered. They then enter wastewater systems and, ultimately, wastewater treatment facilities [4,5,6]. Certain compounds or their metabolites often escape complete elimination within wastewater treatment systems, resulting in significant concentrations discharged from these facilities [7]. The release of treated wastewater into the environment introduces these compounds into various aquatic ecosystems, leading to the presence of antibiotics in concentrations ranging from nanograms per liter (ng/L) to micrograms per liter (µg/L) in rivers, lakes, groundwater, and soils [8,9].

Wastewater treatment plants (WWTPs) have historically focused on removing carbon compounds, inadvertently allowing antibiotics to pass through effluents into water bodies, exacerbating environmental contamination [10,11]. This discharge, coupled with improper disposal practices by pharmaceutical manufacturing industries, hospitals, pharmacies, and households, contributes to the widespread presence of antibiotics in the environment [12,13].

Global antibiotic consumption, estimated at 100,000 to 200,000 tons annually, has led to their detection in WWTP effluents, surface waters, and sediments worldwide, fueling the development of ABR [14,15]. In addition, it is worth noting that most pharmaceutical manufacturing companies disregard local environmental regulations, leading to the discharge of highly contaminated industrial wastewater directly into natural water bodies like streams and rivers, without adequate treatment [16,17]. Additionally, numerous unused or expired medications stored in hospitals, pharmacies, nursing homes, and doctors’ offices are often disposed of by flushing them down the toilet [18,19]. Consequently, antibiotics find their way into both hospital and municipal WWTPs, further contributing to environmental contamination. Wastewater stands out as a critical environment affected by antibiotics due to its role as a convergence point for waste from diverse sources, facilitating the dissemination of ABR [20]. The presence of antibiotics in the environment, even at low concentrations, can have profound adverse effects. Antibiotics and their metabolites can further interact with microbiota, altering composition and metabolism and promoting ABR [21]. Thus, development of ABR among bacteria can further lead to the proliferation of antibiotic-resistant genes.

The global consumption of antibiotics, particularly broad-spectrum penicillin, cephalosporin, quinolones, and macrolides has risen significantly between 2000 and 2015 [22,23]. This global consumption, coupled with occasional misuse, has precipitated the rise of ABR, now recognized as one of the foremost threats to public health in the twenty-first century. The occurrence and concentration of antibiotics in wastewater vary widely, influenced by factors such as geographical region, seasonal changes, demographics, and economics [24]. Given these concerns, it is crucial to determine the concentrations of antibiotics in environmental media and identify potential risks associated with their residues. However, analyzing these residues is challenging due to their low concentrations in complex matrices. Therefore, the development of analytical methods for their detection is essential. Typically, a pre-concentration step using solid-phase extraction (SPE), followed by liquid chromatography (LC) and mass spectrometry (MS) or tandem mass spectrometry (MS/MS), is employed for the determination of pharmaceuticals in wastewater.

Our study represents a pioneering effort, bridging a crucial gap in understanding antibiotic contamination across diverse wastewater systems (WWTPs, hospital wastewater and residential community wastewater) in United Arab Emirates (UAE). By utilizing SPE and advanced LC-MS/MS techniques, we aimed to quantitatively access the diversity, distribution, and prevalence of multiple classes of antibiotics in different types of wastewater sources in UAE. Further, we optimized the conditions for SPE to detect various antibiotics in different types of wastewater sources. Outcome of the study highlights the urgent need for targeted interventions to mitigate the spread of antibiotic resistance, offering invaluable insights for policymakers, environmentalists, and healthcare professionals in safeguarding both human health and environmental integrity.

## 2. Results

### 2.1. Antibiotics Distribution and Quantities in Residential Community, Hospital, and WWTP Wastewater Sources

**Figure 1:**
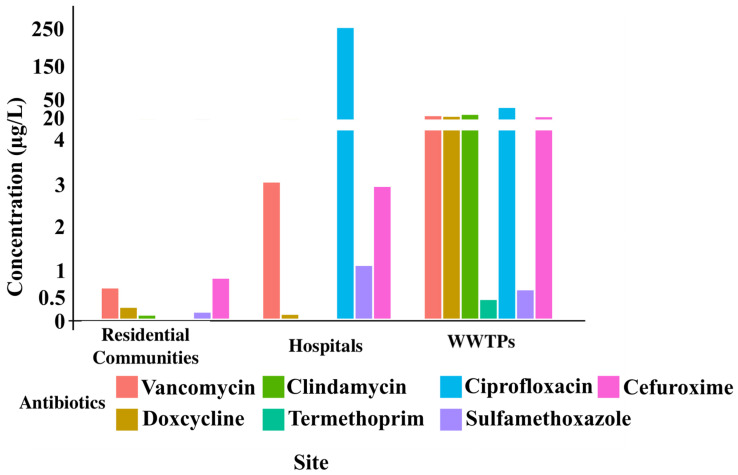
Concentrations of antibiotics in wastewater samples from residential communities, hospitals, and wastewater treatment plants (WWTPs) in the UAE. The figure shows the measured levels (ng/mL) of each antibiotic across the different sampling sites, highlighting variations in occurrence and prevalence.

Antibiotics were quantified in collected samples using reversed-phase capillary ultra-performance liquid chromatography (UPLC) with a double-gradient (pH and organic solvent). Overall, cefuroxime, vancomycin, and ciprofloxacin were the most abundant antibiotics detected (Figure 1). Ciprofloxacin concentrations were notably higher in hospital samples, reaching up to 247.9 ng/mL (Hospital 2; Table S3). Hospital wastewater was often dominated by a single antibiotic, whereas residential community and WWTP samples contained multiple antibiotics at comparable levels.

The ANOVA results revealed non-significant differences in antibiotic concentrations across the three wastewater sources (Table S1), suggesting relatively consistent antibiotic patterns overall. However, within-source comparisons showed significant variation in antibiotic concentrations for hospitals (F-value = 5.59; *p*-value = 0.035) and residential communities (F-value = 10.52; *p*-value < 0.001). When comparing all three sources collectively, significant variation was observed (F-value = 10.52; *p*-value < 0.001; Table S2).

### 2.2. Comparison of Antibiotic Significance in Different Wastewater Types

**Figure 2:**
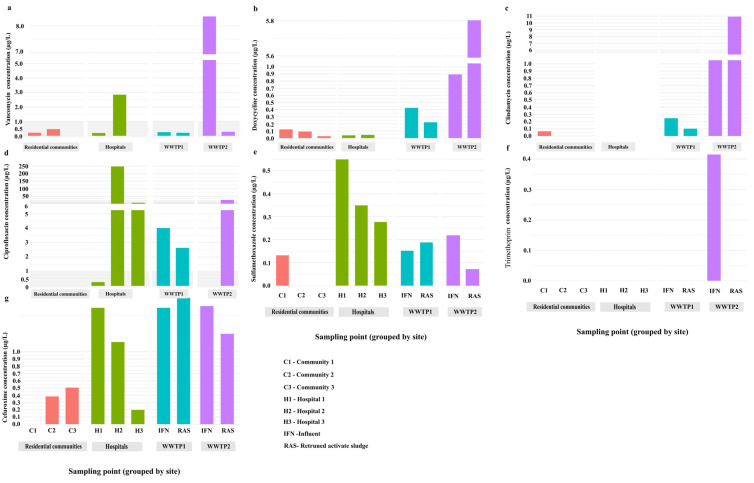
Distribution patterns of antibiotics in hospital wastewater, residential community wastewater, and WWTP samples in the UAE. (**a**) Vancomycin (**b**) Doxycycline, (**c**) Clindamycin, (**d**) Ciprofloxacin, (**e**) Sulfamethoxazole, (**f**) Trimethoprim and (**g**) Cefuroxime. The figure illustrates the relative concentrations of each antibiotic, emphasizing their variability among sources.

Antibiotic concentrations varied significantly across the three wastewater sources (Figure 2). Clindamycin was significantly higher in WWTP samples, while vancomycin and cefuroxime were higher in hospital samples. Ciprofloxacin and sulfamethoxazole levels were also elevated in hospital wastewater compared to residential communities and WWTPs. No significant differences were observed for doxycycline, trimethoprim, and clindamycin between residential and hospital samples.

## 3. Discussion

The observed variability in antibiotic concentrations within hospitals and residential communities highlights the influence of localized antibiotic usage patterns and wastewater discharge practices. The higher ciprofloxacin concentration reaching up to 247.9 ng/mL in hospital wastewater may reflect its frequent prescription for infections Based on a UAE hospital survey, ciprofloxacin and levofloxacin accounted for 88.5% of all fluoroquinolone prescriptions, supporting the higher ciprofloxacin concentrations observed in hospital wastewater and reflecting its frequent use for infections such as urinary tract infections, pneumonia, and bone/joint infections [25,26]. High patient loads, prolonged therapy, and direct discharge from wards may further amplify these levels. Compared with reports from other countries, where hospital effluent ciprofloxacin concentrations are typically in the range of 0.1–10 µg/L, our findings highlight the influence of local prescribing patterns and hospital discharge practices, emphasizing the need for targeted wastewater monitoring and management to mitigate potential environmental and public health risks [27]. Its structural stability, low biodegradability, and strong sorption potential likely contribute to its persistence in the environment and accumulation in soil [28].

The ANOVA results revealed significant within-source variation for hospitals (F = 5.59, *p* = 0.035) and residential communities (F = 10.52, *p* < 0.001), indicating that specific antibiotics differ markedly in concentration across sampling points within the same type of wastewater. For example, ciprofloxacin and sulfamethoxazole were significantly higher in hospital samples, whereas clindamycin was significantly elevated in WWTP samples. In contrast, no significant differences were observed for doxycycline and trimethoprim between residential and hospital samples. These statistical outcomes confirm that localized antibiotic usage and discharge practices directly influence antibiotic profiles within each wastewater source.

While a formal ecological risk assessment using Risk Quotients (RQ = MEC/PNEC) was beyond the scope of this study due to limited site-specific toxicity data, the high ciprofloxacin concentrations detected suggest potential risks to aquatic organisms if discharged untreated. Future studies should perform RQ-based assessments to quantify ecological risks and guide targeted interventions for wastewater management.

The significant differences in clindamycin, vancomycin, and cefuroxime across sources indicate differing selective pressures that could promote resistance development in wastewater microbiota. Elevated clindamycin levels in WWTP samples align with earlier reports from the Middle East region linking this antibiotic to increased resistance prevalence [29]. Similarly, higher levels of vancomycin and cefuroxime in hospital wastewater reflect greater exposure and selective pressure within hospital settings [30,31].

From a methodological perspective, the use of reversed-phase capillary UPLC with a double-gradient provided effective separation of antibiotics, consistent with prior reports validating this approach [32]. However, electrospray ionization requires volatile buffers and reduced salt concentrations, which may introduce challenges such as increased sprayer voltage and potential corona discharge, as previously noted [33].

The persistence of antibiotics such as ciprofloxacin poses a major concern as even low concentrations can facilitate gradual resistance selection among microbial populations [34,35]. Both low and high antibiotic concentrations disrupt microbial communities: high levels eradicate sensitive microbes, leaving behind resistant strains, while low levels drive the stepwise development of resistance [36,37]. Over time, this could transform wastewater streams into reservoirs of multidrug-resistant bacteria, posing risks to human and animal health.

Mitigation strategies should include stricter regulations on effluent treatment, targeted monitoring of high-risk antibiotics, and integration of antimicrobial stewardship programs to reduce antibiotic loading into wastewater systems. Such interventions are critical to curb the spread of AMR and protect environmental and public health.

## 4. Methodology

### 4.1. Reagents and Materials

Standards of antibiotics (based on availability of major antibiotics in wastewater) were purchased from Sigma Aldrich (St. Louis, MO, USA), listed in Table 1. The selected antibiotics represent major therapeutic classes frequently prescribed and commonly detected in wastewater systems. Their inclusion was based on high clinical usage in the UAE, environmental persistence, and analytical suitability for detection and quantification using LC–MS/MS. Oasis HLB cartridges (Oasis HLB 6 cc Vac Cartridge, 500 mg Sorbent per Cartridge) were supplied by Waters (Milford, CT, USA). LC-MS grade Methanol, Formic acid obtained from Fluka (Buchs, Switzerland). Milli-Q-Water was obtained in-house (UAE university). Stocks and working solutions were stored at −20 °C.

### 4.2. Sample Collection

We collected samples from different wastewater sources across the UAE, including hospital wastewater, residential community wastewater, and WWTPs. All samples were collected as grab samples in one-liter sterilized bottles during morning hours (8:00–10:00 AM) to capture typical peak discharge periods. For hospital wastewater, replicated one-liter samples were obtained from sewerage outlets of three different hospitals (*n* = 6). For residential community wastewater, duplicate one-liter samples were collected from the common exit sewerage lines of three distinct communities (*n* = 6). For the WWTPs, two facilities were selected, and samples were collected from influent (Inf1 and Inf2) and returned activated sludge (RAS1 and RAS2) (*n* = 4), consistently during morning hours across sampling days. All samples were immediately transported to the laboratory for processing. In this study, the term “prevalence” refers to the frequency of detection of each antibiotic across the analyzed samples, providing an initial assessment of their occurrence in different wastewater types.

### 4.3. Solid-Phase Extraction (SPE)

To begin the SPE protocol, 1 L of wastewater samples, was collected in solvent-rinsed 1-L amber glass bottles. These bottles were rinsed sequentially with acetone and hexane (1:1 ratio) and then with methanol, using 5 mL of each solvent for rinsing. Following collection, the pH of the sample was adjusted to 2 by adding concentrated HCl dropwise using a glass Pasteur pipette, with pH verification conducted using a pH meter. Then the samples were filtered using Whatman filter paper 41 (Whatman^®^ quantitative filter paper, ashless, Grade 41, Sigma Aldrich, St. Louis, MO, USA). The samples were promptly transported back to the laboratory for further processing, with extraction initiated within 4–6 h of collection.

A glass filtration unit equipped with a 47 mm AP20 glass fiber filter was assembled. The filtration unit was connected to a vacuum inlet, and the sample was passed through the filtration unit under vacuum in increments of 300 mL. Filter replacement was performed as needed based on the sample volume.

For the pre-conditioning of SPE cartridges (Oasis HLB 6 cc Vac Cartridge, 500 mg Sorbent per Cartridge, Waters, Milford, MA, USA), the cartridges were loaded onto the SPE manifold, and vacuum valves were opened. Using a glass serological pipette, 5 mL of acetone and hexane (1:1) were added to the reservoir of each cartridge, allowing the solvent to pass through the sorbent bed by gravity. Additional solvent was added just before reaching the top frit. This process was repeated using methanol and distilled water. Once the cartridges were conditioned, they were ready for extraction.

During the extraction phase, the SPE manifold was connected to a vacuum trap and the vacuum inlet. The adapter of the large volume sampler was connected to the top of the SPE cartridge, ensuring a tight seal, and the weight at the opposite end of the tube was submerged into the sample. Vacuum valves for all samples were opened, and vacuum pressure was applied gently. Adjustments were made as necessary to achieve optimal flow, ensuring that the sorbent bed did not run dry. Once the entire sample had passed through the SPE cartridge, the large volume sampler was disconnected, and the cartridge was left on the manifold (with vacuum) to dry for 2–3 h or until the sorbent was completely dry. After drying, the cartridge was wrapped in aluminum foil and stored appropriately.

### 4.4. Elution

To begin the elution process, the SPE manifold was prepared with the tube rack insert, with a 16 × 150 mm test tube allocated for each sample. Cartridges were loaded onto the SPE manifold, positioned above the appropriate valves for elution into the test tubes. The vacuum valve of each cartridge was then turned on, and 5 mL of methanol was added to the reservoir of each cartridge. The solvent percolated through the sorbent bed and eluted by gravity. This step was repeated once with an additional 5 mL of solvent. Once most of the methanol had eluted, the SPE manifold was connected to the vacuum inlet, and the vacuum was gently turned on to remove most of the solvent from the sorbent bed in approximately 1–2 min. Following this, 5 mL of acetone and hexane (1:1) was added to the reservoir of each cartridge, and the solvent was allowed to percolate and elute by gravity. This step was repeated once with an additional 5 mL of solvent, and then the vacuum was turned back on to remove the remaining solvent from the sorbent bed. Once all the solvent had eluted, the samples were ready to be evaporated.

To evaporate the samples, the test tubes containing the eluted sample were loaded into the nitrogen blow-down unit. The needles of the blow-down unit were lowered into the test tubes, and the nitrogen flow was turned on to create a gentle flow on the surface of the samples, ensuring not to cause the sample to splash around. The needles were lowered every 30 min to maintain a gentle flow on the surface of the samples, taking approximately 1–2 h to blow the samples to dryness. Once completely dry, the tubes were removed from the blow-down unit, and each sample was reconstituted by adding 1000 μL of methanol to each tube. The samples were vortexed thoroughly.

### 4.5. LC-MS/MS

Coupling LC to high-resolution hybrid quadrupole-Orbitrap mass spectrometry enables the identification and quantification of compounds in a single chromatographic run. Parallel reaction monitoring (PRM) scan mode was employed, isolating targeted precursors in Q1 and recording all generated MS/MS fragment ions in parallel, providing accurate mass and high-resolution detection. Antibiotics were baseline separated, and commercial standards were used for quantification.

Matrix-matched calibration curves were constructed with eight points using least-square linear regression (R ≥ 0.998). Recovery was assessed by spiking three concentrations (0.01, 0.05, 0.1 ng L^−1^) into each wastewater matrix, and instrumental repeatability was verified with six consecutive injections of standard solutions. All recoveries were within acceptable ranges according to standard SPE-LC-MS/MS protocols, confirming method accuracy and reproducibility. Blank samples were also analyzed to avoid overestimations.

Instrumental detection limits (ILODs), method limits of detection (LODs), and quantification (LOQs) were determined based on signal-to-noise ratios of 3 and 10, respectively, using spiked samples. LODs and LOQs were calculated using the standard deviation of the lowest calibration point (analyzed 20 times) divided by the slope and multiplied by 3 and 10, respectively.

LC-MS/MS analysis was performed using a Shimadzu LC-30AD (Nexera X2) binary pump and LCMS-8060 Shimadzu mass spectrometer (Shimadzu, Tokyo, Japan). Separation was achieved on a Zorbax Eclipse Plus C18 column (100 mm × 4.6 mm, 3.5 µm) with an injection volume of 10 µL. The autosampler was maintained at 5 °C and the column at 30 °C. The mass spectrometer operated in both positive and negative ESI modes, using argon as the heating and drying gas. The mobile phase consisted of methanol and 0.1% formic acid in water (70:30, *v*/*v*), with rinse volumes of 1000 µL before and after aspiration. The total runtime for analysis was 5 min, with retention times and MS/MS parameters for each antibiotic presented in Table 2. The method was validated in terms of linearity range (Table 3).

From a methodological perspective, the use of reversed-phase capillary UPLC with a double-gradient elution provided effective separation of the target antibiotics, ensuring accurate identification and quantification. The electrospray ionization (ESI) source enabled sensitive detection in both positive and negative ion modes, although it requires volatile buffers and low salt concentrations to avoid issues such as increased sprayer voltage or potential corona discharge. This approach is consistent with prior reports validating UPLC-ESI for trace-level analysis of antibiotics in complex water matrices.

Detection limits, recovery, and repeatability: Instrumental limits of detection (ILODs) and method limits of detection (LODs) and quantification (LOQs) were determined experimentally. LODs and LOQs were calculated from spiked wastewater samples using a signal-to-noise ratio of 3 and 10, respectively, based on 20 replicate analyses of the lowest calibration point. Recovery rates were assessed by spiking three concentrations of each antibiotic (0.01, 0.05, 0.1 ng L^−1^) into each water matrix, followed by SPE and LC–MS/MS analysis. Recoveries for all antibiotics were satisfactory, demonstrating the method’s reliability. Instrumental repeatability was verified using six consecutive injections of standard solutions.

### 4.6. Statistical Analysis

The statistical analyses were conducted using R (v4.0.5). The data was square root transformed before analyses to increase the homogeneity and ANOVA test was performed to determine the significance of antibiotics prevalence in residential community, hospital and WWTP samples. A *p*-value of 0.05 was selected (*p*-value < 0.05 for significant differences). Similar analyses were also performed to compare types of major antibiotics in all three wastewater types and among same wastewater sources for different antibiotics.

## 5. Conclusions

This study provides the first systematic monitoring of antibiotics across diverse wastewater types in the UAE. We have utilized LC-MS/MS-based method, we identify and assess the prevalence of antibiotic across various wastewater sources (WWTP, hospitals and residential community wastewater). High concentrations of cefuroxime and ciprofloxacin were detected, likely reflecting unregulated use and discharge of untreated wastewater. Persistent antibiotic residues indicate incomplete degradation, highlighting the potential role of wastewater as a vector for antimicrobial resistance. Further research is needed to assess environmental risks, the spread of resistance genes, and impacts on soil and crop productivity.

## Figures and Tables

**Table 1:** List of selected antibiotics analyzed in this study and their corresponding information.

Antibiotic Name	Class	Supplier
Amoxicillin trihydrate	β-lactam antibiotic, penicillin	Sigma
Phenoxymethylpenicillin	β-lactam antibiotic, penicillin	Sigma
Cefuroxime sodium	β-lactam antibiotic, cephalosporin	Sigma
Azithromycin	Macrolide antibiotic	Sigma
Clarithromycin	Macrolide antibiotic	Sigma
Ciprofloxacin	Fluoroquinolone antibiotic	Sigma
Clindamycin hydrochloride	Lincosamide antibiotic	Sigma
Doxycycline hyclate	Tetracycline antibiotic	Sigma
Sulfamethoxazole	Sulfonamide bacteriostatic antibiotic	Sigma
Trimethoprim	Dihydrofolate reductase inhibitor	Sigma
Vancomycin hydrochloride	Glycopeptide antibiotic	Sigma

**Table 2:** **Analytical parameters for the targeted antibiotics using LC–MS/MS.** The table includes retention time, precursor and product ion *m*/*z* values, dwell time, quadrupole pre-bias voltages, collision energy (CE), and ionization mode for each compound.

S.No.	Compound Name	Retention Time	Precursor (*m*/*z*)	Product (*m*/*z*)	Dwell Time (ms)	Q1 Pre Bias (V)	CE	Q3 Pre Bias (V)	Mode of Ionization
1	Vancomycin	1.583	725.40	100.10	100	−30	−38	−18	Positive
			725.40	144.15	100	−30	−16	−13	
			725.40	83.10	100	−30	−32	−17	
2	Amoxicillin	1.760	366.00	349.05	100	−30	−10	−24	Positive
			366.00	114.00	100	−16	−21	−22	
			366.00	208.10	100	−10	−13	−13	
3	Doxycycline	2.299	445.05	428.10	100	−19	−20	−20	Positive
			445.05	321.05	100	−21	−31	−21	
			445.05	410.05	100	−11	−25	−28	
4	Clarithromycin	3.172	749.35	158.10	100	−20	−31	−15	Positive
			749.35	591.30	100	−20	−28	−28	
			749.35	116.15	100	−20	−45	−11	
5	Azithromycin	3.177	749.50	158.10	100	−20	−32	−15	Positive
			749.50	591.40	100	−20	−29	−28	
			749.50	116.15	100	−20	−45	−11	
6	Clindamycin	2.154	425.50	126.15	100	−11	−26	−23	Positive
			425.50	377.10	100	−11	−19	−17	
7	Trimethoprim	1.811	291.10	230.10	100	−13	−24	−22	Positive
			291.10	261.05	100	−13	−25	−27	
			291.10	123.05	100	−13	−24	−24	
8	Ciprofloxacin	1.889	332.05	314.10	100	−15	−22	−14	Positive
			332.05	288.10	100	−15	−18	−13	
			332.05	231.05	100	−16	−37	−23	
9	Sulfamethoxazole	2.143	254.00	92.00	100	−12	−27	−19	Positive
			254.00	156.00	100	−12	−16	−15	
			254.00	65.10	100	−12	−47	−25	
10	Phenoxymethylpenicillin	3.621	351.20	160.05	100	−15	−13	−15	Positive
			351.20	114.00	100	−15	−34	−22	
			351.20	87.05	100	−12	−46	−17	
11	Cefuroxime	1.995	423.10	207.15	100	21	14	12	Negative
			423.10	318.05	100	21	9	10	
			423.10	284.10	100	21	16	18	

**Table 3:** **Linearity ranges for the targeted antibiotics, presented in ng/mL.** The ranges indicate the concentrations over which the relationship between the antibiotic concentration and the measured LC–MS/MS response remains linear.

S.No.	Compound Name	Linearity Range Concentration (ng/mL)
1	Vancomycin	0.164 to 26.316
2	Amoxicillin	0.148 to 23.636
3	Doxycycline	0.062 to 9.845
4	Clarithromycin	0.062 to 9.990
5	Azithromycin	0.062 to 9.900
6	Clindamycin	0.03 to 4.766
7	Trimethoprim	0.032 to 5.063
8	Ciprofloxacin	0.052 to 8.25
9	Sulfamethoxazole	0.076 to 12.206
10	Phenoxymethylpenicillin	0.155 to 24.863
11	Cefuroxime	22.995

## Data Availability

The original contributions presented in this study are included in the article/Supplementary Materials. Further inquiries can be directed to the corresponding author.

## Supplementary Materials

The following supporting information can be downloaded at https://www.mdpi.com/article/10.3390/antibiotics14111105/s1. Table S1. AVOVA summary for wastewater treatment plants, hospitals, and residential communities’ samples. Table S2. ANOVA test comparison between and among wastewater treatment plants’, hospitals’, and residential communities’ samples. Table S3. Antibiotics concentration (ng/mL) in three different wastewater types (residential communities, hospitals, and wastewater treatment) plants collected across UAE.
