# Supplementary material for: Environmental Footprint of Antibiotics: A Multi-Source Investigation of Wastewater Systems in UAE
Source: Antibiotics (Basel). 2025 Nov 2;14(11):1105. doi: 10.3390/antibiotics14111105 (PMC12649524; doi:10.3390/antibiotics14111105)
Supplement: Supplementary file 1 [file antibiotics-14-01105-s001.zip › antibiotics-3940846-supplementary.pdf]

Environmental Footprint of Antibiotics: A Multi-Source Investigation of Wastewater Systems in UAE

Shahana Seher Malik [1], Balamurugan Sadaiappan [1,2], Ashraf Aly Hassan [3], Iltaf Shah [4], Sampathkumar Elangovan [4] and Sunil Mundra [1,2,5,*]

1 Department of Biology, College of Science, United Arab Emirates University, Al Ain P.O. Box 15551,

United Arab Emirates; 202090120@uaeu.ac.ae (S.S.M.); balamurugan.s@uaeu.ac.ae (B.S.)

2 Khalifa Center for Genetic Engineering and Biotechnology, United Arab Emirates University,

Al Ain P.O. Box 15551, United Arab Emirates

3 Department of Civil and Environmental Engineering, College of Engineering, United Arab Emirates University, Al Ain P.O. Box 15551, United Arab Emirates; alyhassan@uaeu.ac.ae

4 Department of Chemistry, College of Science, United Arab Emirates University, Al Ain P.O. Box 15551, United Arab Emirates; altafshah@uaeu.ac.ae (I.S.)

5 National Water and Energy Center, United Arab Emirates University, Al Ain P.O. Box 15551,

United Arab Emirates

* Correspondence: sunilmundra@uaeu.ac.ae; Tel.: +971-7136341

Supplementary information: One file containing three Tables (Table S1 to Table S3)

Table S1: AVOVA summary for wastewater treatment plants, hospitals, and residential communities' samples.

**Sampling Site**	**Sum of Squares (SS)**	**Mean Square (MS)**	**Degrees of Freedom (DF)**	**F-value**	**p-value**
Wastewater treatment plants	0.02583	0.01292	2	1.05	0.399
Hospitals	0.6906	0.0987	2	5.594	0.0354
Residential communities	0.18981	0.09491	2	10.52	0.000778

Table S2: ANOVA test comparison between and among wastewater treatment plants', hospitals', and residential communities' samples.

**Source of Variation**	**Sum of Squares (SS)**	**Degrees of Freedom (df)**	**Mean Square (MS)**	**Factor F**
Between groups	8.81	2	4.405	0.000009679
Within groups	365,995,340.19	7	52,285,048.60	
Total	365,995,349	9		

Table S3: Antibiotics concentration (ng/mL) in three different wastewater types (residential communities, hospitals, and wastewater treatment) plants collected across UAE.

Sites	Sampling point	Antibiotics						
		**Vancomycin**	**Doxycycline**	**Clindamycin**	**Trimethoprim**	**Ciprofloxacin**	**Sulfamethoxazole**	**Cefuroxime**
**Residential communities**	**Community 1**	**0.212**	**0.122**	**0.065**	0.000	0.000	**0.132**	0.000
	**Community 2**	**0.460**	**0.092**	0.000	0.000	0.000	0.000	**0.384**
	**Community 3**	0.000	**0.026**	0.000	0.000	0.000	0.000	**0.506**
**Hospitals**	**Hospital 1**	**0.188**	**0.039**	0.000	0.000	**0.260**	**0.549**	**1.607**
	**Hospital 2**	**2.848**	**0.045**	0.000	0.000	**247.926**	**0.349**	**1.134**
	**Hospital 3**	0.000	0.000	0.000	0.000	**6.317**	**0.277**	**0.198**
**WWTP1**	**Influent**	**0.252**	**0.425**	**0.246**	0.000	**4.008**	**0.152**	**1.607**
	**Returned activated sludge**	**0.207**	**0.222**	**0.101**	0.000	**2.634**	**0.188**	**1.746**
**WWTP2**	**Influent**	**8.354**	**0.895**	**1.491**	**0.415**	0.000	**0.219**	**1.631**
	**Returned activated sludge**	**0.275**	**5.803**	**10.928**	0.000	**25.321**	**0.072**	**1.248**
